# Effects of Expectancy on Cognitive Performance, Mood, and Psychophysiology in Healthy Adolescents and Their Parents in an Experimental Study

**DOI:** 10.3389/fpsyt.2020.00213

**Published:** 2020-03-17

**Authors:** Daniel Watolla, Nazar Mazurak, Sascha Gruss, Marco D. Gulewitsch, Juliane Schwille-Kiuntke, Helene Sauer, Paul Enck, Katja Weimer

**Affiliations:** ^1^Department of Psychosomatic Medicine and Psychotherapy, University Medical Hospital Tübingen, Tübingen, Germany; ^2^Department of Psychosomatic Medicine and Psychotherapy, Medical Psychology, Ulm University Medical Center, Ulm, Germany; ^3^Department of Psychology, Clinical Psychology and Psychotherapy, University of Tübingen, Tübingen, Germany; ^4^Institute of Occupational and Social Medicine and Health Services Research, University Hospital Tübingen, Tübingen, Germany; ^5^Department of Psychosomatic Medicine and Psychotherapy, Ulm University Medical Center, Ulm, Germany

**Keywords:** placebo effect, expectancy, cognitive performance, mood, heart rate variability, skin conductance

## Abstract

**Objective:**

Placebo effects on cognitive performance and mood and their underlying mechanisms have rarely been investigated in adolescents. Therefore, the following hypotheses were investigated with an experimental paradigm: (1) placebo effects could be larger in adolescents than in adults, (2) parents’ expectations influence their adolescents’ expectations and placebo effects, and (3) a decrease in stress levels could be an underlying mechanism of placebo effects.

**Methods:**

Twenty-six healthy adolescents (13.8 ± 1.6 years, 14 girls) each with a parent (45.5 ± 4.2 years, 17 mothers) took part in an experimental within-subjects study. On two occasions, a transdermal patch was applied to their hips and they received an envelope containing either the information that it is a Ginkgo patch to improve cognitive performance and mood, or it is an inactive placebo patch, in counterbalanced order. Cognitive performance and mood were assessed with a parametric Go/No-Go task (PGNG), a modification of California Verbal Learning Test, and Profile of Mood Scales (POMS). Subjects rated their expectations about Ginkgo’s effects before patch application as well as their subjective assessment of its effects after the tests. An electrocardiogram and skin conductance levels (SCLs) were recorded and root mean square of successive differences (RMSSD), high-frequency power (HF), and the area under the curve of the SCL (AUC) were analyzed as psychophysiological stress markers.

**Results:**

Expectations did not differ between adolescents and parents and were correlated concerning reaction times only. Overall, expectations did not influence placebo effects. There was only one significant placebo effect on the percentage of correct inhibited trials in one level of the PGNG in adolescents, but not in parents. RMSSD and HF significantly increased, and AUC decreased from pre- to post-patch application in adolescents, but not in parents.

**Conclusion:**

With this experimental paradigm, we could not induce relevant placebo effects in adolescents and parents. This could be due to aspects of the study design such as application form and substance, and that healthy subjects were employed. Nevertheless, we could show that adolescents are more sensitive to psychophysiological reactions related with interventions which could be part of the underlying mechanisms of placebo effects in adolescents.

## Introduction

The term “placebo effect” can be described as a symptom improving effect of a drug without an active agent; for example in the context of placebo-controlled, randomized clinical trials (RCTs). A placebo response is defined as the effectiveness of a placebo on symptoms in the context of RCTs, whereas the placebo effect is part of a symptom change, which can be directly attributed to placebo mechanisms such as expectations or learning mechanisms after eliminating external unspecific factors and statistical artifacts (1, 2). The placebo response is well documented and robust effects have been replicated especially in placebo analgesia (1). To date many aspects concerning the placebo response and effect have been discovered, for example mechanisms, mediators and moderators (1, 3). In a recent review about factors predicting placebo responses, it was concluded that placebo responses mainly appear to be moderated by expectations of how the symptom might change after treatment, or expectations of how symptom repetition can be coped with (4). A handful of moderators—circumstances under which placebo effects occur—have been discussed, among them are age, sex, and personality traits (4, 5).

Beyond the numerous findings in the context of pain reduction, the question arises whether there are also placebo effects on mood, emotional states, and cognitive performance. Concerning placebo analgesia, the reduction of negative emotions mediating pain reduction, rather than the placebo effect reducing pain directly (6, 7) has been analyzed. The discussion is supported by findings of a reduction of electrophysiological stress markers such as heart rate variability (HRV) and subjective stress by an experimental placebo intervention on heat pain (8). There was a decrease in the HRV low-frequency (LF)/high-frequency (HF) ratio after placebo administration but not in the control group, which was interpreted as a decrease in sympathetic activation indicating lower stress levels. In a regression analysis, subjective stress was the only significant predictor of the placebo effect on pain reduction. Subjective stress itself was only significantly predicted by LF/HF ratio decrease and subjective mood. Another study of this group showed that placebo administration could decrease anticipatory stress which was correlated with placebo analgesia (9). These findings support the hypothesis that placebo effects could alter stress levels and negative emotions, which are, in turn, able to mediate the effects of pain reduction. It needs to be further studied whether this also applies to situations outside the context of pain reduction.

In experimental studies, placebo effects on mood and emotions were previously investigated in the context of pain, but only rarely with regard to depression, a negatively altered pathological state of mood and emotionality. Factors influencing the placebo effect on depression have been investigated through meta- and re-analyses of RCTs [see for example (3, 10, 11)]. There is evidence that neurobiological mechanisms produce placebo effects on mood and behavior, such as an opioid and dopamine modulation of the hypothalamic-pituitary-adrenal axis (12). Considering these results for mood improvement after placebo intake in the clinical context of depression, the question arises, whether and under which circumstances these kinds of effects also appear in a healthy population, and whether they can be experimentally induced. For example, some recent experimental studies measured mood with the Profile of Mood State Questionnaire (POMS) (13) or other affective state scores and examined the effects of placebo interventions in healthy populations (14–18).

Contrary to placebo effects in the context of pain, relatively little is known about placebo effects on cognitive performance. These effects are often examined in the context of substance (ab)use, with users hoping to benefit from the positive effects, for example on aspects of cognitive performance like memory or concentration. Beyond the physiological effects of a substance, placebo effects seem to play an important role in affecting behavior and cognitive performance. With regard to cognitive effects, methylphenidate is an increasingly used substance for “cognitive enhancement,” not only in clinical use (for example for attention-deficit/hyperactivity disorder, or ADHD) but also in non-medical use by healthy people (14). A recent study involving Swiss school students with an average age of 17.1 years found a lifetime prevalence of almost 55% in substance abuse for cognitive enhancement, and a 13.3% lifetime prevalence for the use of prescription or recreational drugs (19). However, the role of stimulants as “cognitive enhancers” has been questioned even as medication (20), as the positive change in symptoms after stimulant treatment of children and adolescents with ADHD seems to be partly related to placebo effects (21). A simple experiment could show that students who responded to a flyer advertising a training for cognitive enhancement performed significantly better in a cognitive task than those who responded to a flyer advertising the same study with the benefit to receive credit points (22). Moreover, contradictory experimental findings in the context of everyday substances such as nicotine and caffeine do exist, with only some of them showing placebo effects on cognitive parameters (5, 15–17, 23).

The placebo effect in children and adolescents has recently been reviewed with the conclusion that only little data exists, and that a relatively low number of studies handled the placebo effect per se in children and adolescents (24). In general, placebo responses in clinical trials tend to be higher in children and adolescents (24). In two of the few experimental studies on the placebo effect in children with similar designs, it was possible to induce placebo effects in healthy children in a heat placebo analgesia design (25, 26). The latter describes their expectancy induced placebo analgesia response as substantially higher than those typically found in adults, yet a control group was not used. Contrary effects have not been more distinct compared to an adult control group (25). This finding raises the question whether the placebo effect in children or adolescents might depend on their disease and developmental state (25, 27). Concerning the mechanisms of placebo effects in children and adolescents, higher learning capacities, associative learning, and learning capacities in general might play a more important role. Furthermore, other forms of learning like social learning or imitation might be more important in children and adolescents with an increased influence from peer groups and media (24). Social learning of placebo effects through observation of a beneficial and successful analgesic treatment was shown in health women, and this treatment was as effective as a conditioning procedure (28). If social learning of placebo effects works in children and adolescents has yet not been investigated. However, children’s or adolescents’ own expectations might play a subordinate role in producing the placebo effect (24, 29). This assumption goes in line with the “placebo by proxy” effect (30), a placebo effect on patients’ environment eventually contributing in turn to symptom improvement in the patient. The research on children’s and adolescents’ placebo effects has consequently begun to arouse interest and should be further investigated with regard to the underlying mechanisms and the dependency on age, developmental state, diseases, expectations, and moderating traits’ influences.

As outlined in *Introduction*, many aspects of the placebo effect, especially outside the pain context, are yet unknown and would be worth investigating, preferably in an experimental study. Thus, the presented study has three goals: (1) the primary objective is to compare healthy adolescents with their parents regarding the experimentally induced placebo effect on mood and cognitive performance—measured *via* psychological questionnaires, reaction, and memory tests. It is hypothesized that placebo effects can be induced by an ineffective alleged Ginkgo transdermal patch, and that this effect is greater in adolescents than in adults. (2) Parents’ expectations about Ginkgo effects influence their children’s expectations and placebo effects and they, therefore, are correlated. (3) Finally, we will exploratively investigate whether this placebo application can decrease stress levels measured as psychophysiological responses such as HRV and skin conductance levels (SCL). We will also analyze if they differ between adolescents and parents.

We therefore performed a study with two experimental sessions following a within-subjects design to induce placebo effects on cognitive performance and mood in parent–child dyads. Effects were induced with help of an inactive transdermal patch accompanied by the information that this patch is either a Ginkgo patch which improves mood and cognitive performance, or it is a non-effective placebo patch. The context of Ginkgo was chosen, because it is assumed that expectations about its effectiveness exist in the general population, as Ginkgo is advertised and sold as having proven positive effects on memory (31). To the authors’ knowledge, a comparable experimental design with adolescents as subjects has never been done before, especially not in comparison to their parents.

## Materials and Methods

### Sample

The subjects were recruited by advertisements at the medical university campus and public places in the city of Tübingen and through mail distribution lists. The advertisement for the study used the pretext of testing the impact of expectancy on the effects of a new Ginkgo preparation and an idea of the procedure was given. Before being invited, a telephone interview was conducted in which the participants’ suitability was checked by ruling out acute or chronic somatic and psychiatric diseases and any mood- or reaction-altering drug use. Applicants who were pregnant or breastfeeding were also ruled out. Only one child parent pair was rejected for not fulfilling the criteria and two further suitable pairs refused further participation after the interview for personal reasons.

All adolescents and parents were included after written informed consent only. This study was approved by the Ethical Review Board of the University of Tübingen (project No. 295/2013BO1) and was conducted in accordance with the Declaration of Helsinki.

### Procedure

The experiment followed a within-subjects design and child–parent pairs were invited to two sessions which took place at the same time of the day with an interval of at least 3 days. All experiments were conducted by the same male investigator (DW) who wore neutral clothing in a research lab. At the beginning of the first session the subjects were handed a written document informing them about the study’s procedure, length, risks, voluntariness, data protection, monetary compensation, and the fact that not all details of the study are revealed to the participants. We therefore followed the concept of “authorized deception” (32). The subjects had to sign a consent form and parents additionally had to sign for their children. There were no refusals.

The general procedure explained in the following sections was identical for both sessions. At the beginning of each session the subjects’ physical condition was examined by measuring blood pressure and heart rate. Furthermore, the participants’ general health was checked as well as if they abstained from alcohol and any drugs during the previous 24 h. Afterwards three electrodes were placed on the chest to record their electrocardiogram (ECG), and two electrodes were attached to their fingers for the assessment of the SCL (see below). A 5-min baseline measure was recorded, followed by the assessment of the POMS (13) baseline measure (pre) and a questionnaire about their expectancies about the possible effects of the Ginkgo preparation on reaction time, concentration, memory, and mood. Expectancies were assessed by the question “How effectively do you think that Ginkgo will affect your reaction time (concentration, memory, or mood, respectively)?” and rated by subjects on a visual analog scale (VAS) from “worsening” through “no change” to “improvement.” The VAS was quantified from −50 to +50 mm for further analyses. After these preparations, the subjects received an envelope in which it stated whether they would get a Ginkgo patch, improving their mood and cognitive performance or a placebo patch, which would not improve their mood and cognitive performance. In fact, they always got a placebo patch which did not contain any active agent. Actually only the information (stimulus expectancy) was changed between the two sessions in a counterbalanced manner so that the Ginkgo information was given in the first or second session. Adolescents and parents were always in the same condition and thus received the same information. The experimenter was kept blind to the order of the conditions: the envelopes with the information were prepared in advance by another person of the lab, and subjects were told, not to tell the content of the envelope at any time in order to keep the experimenter blind. The exact wording in the envelope was according to the condition: “Today you are going to get a Ginkgo (placebo) patch. So, you are in the experimental (control) condition. Don’t tell the experimenter about the today’s condition during the experiment.” After the subjects received their information the experimenter fixed the approx. 5 × 7.5 cm transdermal patches on the participants’ hips. From then on, parents and adolescents were separated in two rooms. They had to wait for approximately 15–20 min after patch application, then POMS was filled out a second time (post) to evaluate mood changes. The cognitive tests began 25–30 min after the patch application.

The first cognitive test conducted was the California Verbal Learning Test (CVLT) (33). Subjects were informed that this is a word memory test. The instruction was read literally (translated from German): “Now I’m going to read a list of words to you. Please learn the words by heart and reproduce them afterwards. I’ll read the list to you just once and the order in which you reproduce the words does not matter.” As soon as the subject was ready, the 10 words were read at a rate of approximately 1 Hz. The subject was then asked to reproduce the words and every correct answer was noted (first recall). The subject did not receive any feedback regarding their accuracy, not even when asked. There was no time limit for reproducing the words. Afterwards, the parametric Go/No-Go task (PGNG) (34) was administered. The instruction was included in the program and every level of the task was explained step by step with examples following a test trial. The test took approximately 15–20 min and the time period was marked on the electrophysiological device. The PGNG ended approximately 45–50 min after patch application, and the second recall phase of the CVLT began. The subject was literally asked (translated from German): “Do you remember the learned words from the list? Please reproduce them. Again, the order does not matter.” Every correct answer was noted (second recall). Immediately after finishing, the third phase of the CVLT began in which the subject had to recognize the 10 words from the list out of a sum of 30 words. The subject was asked (translated from German): “Which of the following words were included in the former list of recalled words? Answer with yes or no.” The experimenter read the list and waited for the subjects’ answer after each word. The answer was written down by the experimenter and again the subject did not get any feedback regarding his/her answers. Without knowing if the word was actually in the list, the subject was advised to go with his gut feeling. All correct words were counted as “hits.” This phase was the last to be registered on the electrophysiological device. Finally, the subjects completed a questionnaire concerning the effectiveness of the patch received on the same VAS as at the beginning for expectations (subjective outcomes). The electrodes and the device were removed. The whole procedure took approximately 1 h. After the second session the family received their payment for participation (20 Euros for the parent and cinema vouchers worth 20 Euros for each participating child) and was informed about the whole experiment; especially about the patches not containing any active agent in both sessions. It was explicitly pointed out that all the administered data could be deleted if desired, but nobody wanted their data to be deleted.

### Measurement of Cognitive Performance, Mood, and Subjective Outcomes

To measure placebo effects on cognitive performance, a PGNG test (34) was used. The PGNG measures reaction time, inhibition, and executive functions. It contains three levels of ascending difficulty, in which single letters are shown rapidly in the middle of a screen. Mean reaction time over correct targets (RTT) and percentage of correct target trials (PCTT) in all three levels, and the percentage of correct inhibitory trials (PCIT) in levels 2 and 3 were analyzed as dependent variables for concentration and reaction times. To test placebo effects on memory, an adaptation of the CVLT (33) was used. The sum of max. 10 words learned by heart and immediately recalled (first recall) as well as the sum of recalled words with delay (second recall) and the correct recognized words (hits) from the list at the end of CVLT were analyzed as dependent variables for memory. To operationalize the hypothesized change of mood, the shortened version of the POMS (13) was used. It contains 19 items to rate current positive and negative emotions, such as joy, anger, depression, fatigue, and tension on a 7-point Likert scale. For further analyses, sums of the POMS positive scale ranging from 6 to 42 points, and the POMS negative scale ranging from 13 to 91 were calculated. Differences between the POMS scales before and after patch application were used as dependent variables (positive values indicate higher values of the scale after the patch application).

To assess subjectively recognized effects of the patches, subjects were asked to fill out a questionnaire concerning the extent of the influence of the patch they received on VASs for reaction time, concentration, memory, and mood, at the end of each experimental session.

### Electrophysiological Data

Electrophysiological data was collected in the form of interbeat intervals (IBIs) and SCLs using a 3991x-GPP BioLog recorder, firmware Version 1.2 (2012). A three channel ECG was set up on the participants’ thoraxes on the level of second intercostal space left and right and below the left mammilla (see *Procedure*). Data was read out and saved by the 3991x-GPP DPS software, Version 1.2 (2012) immediately after each session. For the analysis of the HRV data, 6 subjects had to be excluded due to technical problems during recording or movement artifacts, resulting in 42 datasets (20 parents, 22 adolescents). The data handling of the HRV data was carried out with Kubios HRV, Version 2.2 using autoregression with a model order of 16 without factorization as spectrum estimation. Trend removal was applied by smoothing priors with lambda = 500. Artifact correction was used stepwise when needed. In 57.1% no artifact correction was used, in 5.9% very low, in 4.6% low, in 30.7% medium, and in 1.7% strong artifact correction was used. The parameters of interest concerning HRV were the root mean square of successive difference (RMSSD) and the logarithmically transformed HF power (0.15–0.4 Hz) in the autoregression spectrum (HF). These two parameters are known to represent vagal influence on HRV (35). In this study these parameters are supposed to reflect a decreased state of stress or arousal. Two 5-min time frames of measurement were chosen: 1) baseline after installation of the device at the beginning of the session, and 2) immediately after patch application while filling out personality questionnaires.

In contrast to HRV, which is a surrogate for parasympathetic activity and reactivity, the SCL represents sympathetic activity and reactivity. SCL is considered to be a good indicator of the “inner tension” of subjects. Two electrodes connected to the BioLog device were positioned on the index and the ring fingers of the non-dominant hand to detect conductivity changes. The SCL signal was detected with a rate of 10 Hz and between 0.1 and 39.9 μMho. Due to the adequate data quality, no other preprocessing steps were necessary, and the mean of the signal (SCL-M) as well as the area under the curve (SCL-AUC) were calculated (36, 37).

### Statistical Analyses

All statistical analyses were performed with IBM SPSS Version 22 (IBM Corp., Armonk, NY). Significance level was set to α = 0.05. Sample size was calculated for the main analyses, the 2 × 2 repeated-measures ANOVA (condition × age group) for which a total sample size of n = 34 was sufficient to detect a medium effect size of f = 0.25 (with *r* = 0.3, α = 0.05, power = 0.80), as calculated with G*Power Version 3.1.9.2 (38). Normal distribution of variables was assessed with Shapiro–Wilk tests and visual inspection of normal quantile–quantile plots. As some expectations were not normally distributed, Mann–Whitney U tests and Spearman correlations were used to analyze differences and associations between adolescents’ and parents’ expectations at first appointment when they were not influenced by any condition assignment, and between parents’ expectations and adolescents’ placebo effects. Placebo effects were calculated as the difference between the Ginkgo and the placebo condition for each outcome. In order to rule out possible sequence effects of the information given (Ginkgo vs. placebo) at the first and second appointment, all presented repeated-measures ANOVAs were rerun with sequence order as an additional factor. There were no main or interaction effects for any of the analyzed dependent variables (results not reported). To investigate whether placebo effects differ between adolescents and parents, 2 × 2 repeated-measures ANOVAs with condition (told placebo vs. told Ginkgo) as within-subjects factor and age group (adolescents vs. parents) as between-subjects factor were performed. As *post hoc* tests, differences between conditions (told placebo vs. told Ginkgo) were tested with paired t-tests for adolescents and parents separately. In order to control for multiple testing *p*-values were adjusted according to Hochberg (39).

With regard to psychophysiology, separate 2 × 2 × 2 repeated-measures ANOVAs were performed with condition (told Ginkgo vs. told placebo) and time point (baseline vs. post-patch) as within-subjects factors and age group (adults vs. adolescents) as between-subjects factor for each of the dependent variables RMSSD, HF, SCL-M, and SCL-AUC.

## Results

### Sample Description

Twenty-six healthy adolescents between 12 and 17 years (13.8 ± 1.6 years; 12 boys, 14 girls) each with a parent (45.5 ± 4.2 years; 5 fathers, 17 mothers of which 4 mothers participated with 2 children) participated in the experiment, leading to a total of 48 subjects (because of four threesomes). Except for one girl, all the adolescents were in a German “Gymnasium,” which is the highest secondary school level. The parents all had at least an education or had graduated. Except of one mother who had already tried homoeopathic Ginkgo sweets, none of the participants reported any experience with Ginkgo products.

### Expectations

At first appointment, expectations of Ginkgo effects did not differ between adolescents and parents in general and were significantly correlated between adolescents and their own parent concerning effects on reaction times only (**Table 1**). Furthermore, there was only one significant correlation between the expectation of the effects on mood and the placebo effect on negative mood in parents (*r* = −0.523, *p* = 0.013, adjusted *p* = 0.156), whereas there was no correlation between expectations and placebo effects in adolescents. Regarding the influence of parents’ expectations on adolescents’ placebo effects, there was one correlation between parents’ expectation of Ginkgo effects on reaction and adolescents’ placebo effect on reaction time in level 3 (*r* = 0.395, *p* = 0.046, adjusted *p* = 0.966).

**Table 1:** Expectations of adolescents and parents concerning the effects of Ginkgo on outcome measures: differences between adolescents and parents in general (Mann–Whitney U tests), and correlation between adolescents and own parents (Spearman correlations) (reported as median [1^st^–3^rd^ quartile]).

	Adolescents	Parents	Mann–Whitney test	Spearman *r*
Concentration	25.0 [21.0–28.0]	20.5 [11.8–32.3]	*Z* = −0.69, *p* = 0.488	*r* = −0.151, *p* = 0.470
Reaction time	20.0 [8.0–24.5]	15.0 [0.0–31.3]	*Z* = −0.08, *p* = 0.940	*r* = −0.469, *p* = 0.018
Memory	12.3 [0.0–28.0]	20.0 [7.3–35.5]	*Z* = −1.64, *p* = 0.101	*r* = −0.154, *p* = 0.472
Mood	0.0 [0.0–4.0]	12.8 [0.0–28.3]	*Z* = −1.84, *p* = 0.065	*r* = −0.099, *p* = 0.636

### Placebo Effects on Cognitive Performance: Reaction Times, Correct Trials, and Memory

The 2 × 2 repeated-measures ANOVAs with condition (told Ginkgo vs. told placebo) as within-subjects factor and age group (adults vs. adolescents) as between-subjects factor showed a signiﬁcant main effect of condition for PCTT level 3 as dependent variable only (*F*(1,46) = 8.91, *p* = 0.005), but without an interaction of condition × age group (*F*(1,46) = 3.48, *p* = 0.069). According to *post hoc* tests, adolescents showed a significantly lower PCTT in level 3 (worse cognitive performance) in the Ginkgo compared to the placebo condition, whereas no other comparison was significant neither in adolescents nor in parents (**Table 2**). The only significant placebo effect was found for PCIT level 2: There was a significant interaction of condition × age group (*F*(1,46) = 9.56, *p* = 0.003) with a higher PCIT in the Ginkgo compared to the placebo condition in adolescents but with nearly no change in parents (**Table 2**). Additionally, there were signiﬁcant effects of the between-subjects factor age group (adults vs. adolescents) in mean reaction times: RTT level 1 (*F*(1,46) = 15.48, *p* < 0.001), RTT level 2 (*F*(1,45) = 35.47, *p* < 0.001), and RTT level 3 (*F*(1,46) = 18.89, *p* < 0.001) indicating faster reaction times in all three levels for adolescents.

**Table 2:** Cognitive performance (PGNG, CVLT) in the told placebo and told Ginkgo conditions in adolescents and parents (mean ± SD).

Outcome	Adolescents				Parents			
	Placebo	Ginkgo	*p*	Adj. *p*	Placebo	Ginkgo	*p*	Adj. *p*
RTT, L1 (ms)	410 ± 21	414 ± 23	0.374	> 0.999	435 ± 26	436 ± 26	0.804	0.825
PCTT, L1 (%)	73.2 ± 16.2	69.9 ± 17.5	0.302	> 0.999	64.9 ± 27.0	63.9 ± 23.2	0.756	0.825
RTT, L2 (ms)	399 ± 19	405 ± 26	0.108	0.648	437 ± 25	439 ± 22	0.668	0.825
PCTT, L2 (%)	76.0 ± 19.5	70.1 ± 20.3	0.084	0.588	58.6 ± 23.8	57.1 ± 25.7	0.707	0.825
PCIT L2 (%)	78.7 ± 13.1	86.7 ± 13.2	0.005	0.05	93.9 ± 5.4	91.8 ± 8.5	0.231	0.825
RTT, L3 (ms)	427 ± 16	434 ± 15.5	0.079	0.588	458 ± 24	453 ± 34	0.483	0.825
PCTT, L3 (%)	53.4 ± 16.4	43.4 ± 15.5	0.003	0.033	32.0 ± 21.4	29.7 ± 20.9	0.394	0.825
PCIT L3 (%)	66.2 ± 21.0	72.5 ± 15.7	0.074	0.588	88.0 ± 12.9	86.7 ± 13.8	0.648	0.825
CVLT, 1^st^ recall	6.12 ± 1.51	6.15 ± 1.46	0.908	> 0.999	6.59 ± 1.22	6.45 ± 1.26	0.710	0.825
CVLT, 2^nd^ recall	4.35 ± 1.83	4.73 ± 1.54	0.210	> 0.999	4.91 ± 1.82	4.68 ± 1.64	0.707	0.825
CVLT, hits	8.38 ± 1.33	8.38 ± 1.27	> 0.999	> 0.999	8.64 ± 1.18	8.68 ± 0.95	0.825	0.825

RTT, reaction time to target; PCTT, percentage correct target trials; PCIT, percentage correct inhibited trials; L, level; CVLT, California Verbal Learning Test; Paired t-tests, adjusted p values according to Hochberg (39).

There was no signiﬁcant placebo effect on memory in any of the three dependent variables of the CVLT, and no difference between age groups or significant interaction (**Table 2**). With regard to the condition as a main effect, the statistics for ﬁrst recall were *F*(1,46) = 0.04, *p* = 0.842, for second recall *F*(1,46) = 0.06, *p* = 0.806, and for hits *F*(1,46) = 0.02, *p* = 0.888.

### Placebo Effects on Mood and Subjective Outcomes

Changes in the POMS scales from pre- to post-patch application for both conditions are reported in **Table 3**. Note that a negative difference indicates a decrease and a positive difference indicates an increase from pre- to post-patch application in the respective mood scale. In both dependent variables there was no signiﬁcant effect in 2 × 2 ANOVAs, neither for the within-subjects factor nor for the between-subjects factor or the interaction. With regard to condition as main effect the statistics for positive emotions were *F*(1,46) = 1.02, *p* = 0.317, for negative emotions they were *F*(1,46) = 1.62, *p* = 0.209. However, adolescents reported significantly better mood in response to the Ginkgo compared to the placebo patch at least according to the unadjusted *p* value.

**Table 3:** Subjective assessments of the effects of the patches in the told placebo and told Ginkgo conditions in adolescents and parents (mean ± SD).

Outcome	Adolescents				Parents			
	Placebo	Ginkgo	*p*	Adj. *p*	Placebo	Ginkgo	*p*	Adj. *p*
POMS negative	−0.35 ± 4.27	−2.15 ± 2.78	0.107	0.428	−0.82 ± 2.34	−0.68 ± 2.25	0.830	0.830
POMS positive	0.04 ± 2.71	1.62 ± 3.02	0.048	0.250	−0.36 ± 3.90	−0.64 ± 3.46	0.803	0.830
Concentration	5.85 ± 11.52	6.17 ± 16.18	0.930	0.930	2.05 ± 5.05	10.38 ± 16.58	0.021	0.126
Reaction time	2.56 ± 10.04	6.63 ± 15.45	0.246	0.688	2.60 ± 4.99	4.40 ± 16.89	0.661	0.830
Memory	0.04 ± 14.42	4.02 ± 16.90	0.344	0.688	3.05 ± 8.70	8.39 ± 19.92	0.292	0.830
Mood	3.65 ± 7.51	8.81 ± 11.99	0.050	0.250	2.40 ± 7.07	8.93 ± 15.54	0.086	0.430

POMS, Profile of Mood Scale; Paired t-tests, adjusted p values according to Hochberg (39).

ANOVAs with the subjective assessments of the effects of the patches on reaction time, concentration, memory, and mood as dependent variables revealed a significant main effect of the condition for mood only (*F*(1,44) = 7.53, *p* = 0.009), with perceived better mood after Ginkgo compared to the placebo condition independent of age group. Post hoc paired t-tests suggest that this effect may consist on adolescents’ assessments only although analyses do not withstand *p*-value adjustment.

### Psychophysiological Data

**Figure 1:**
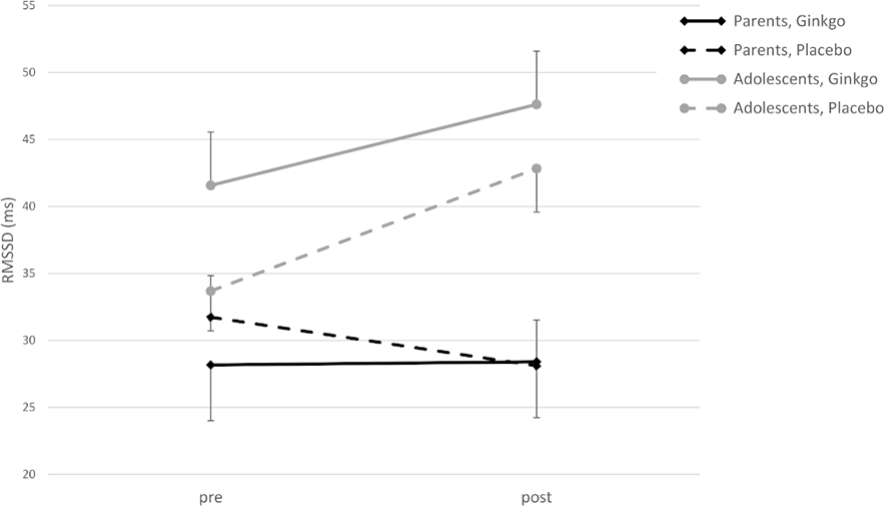
Root mean square of successive differences (RMSSD) (ms) in adolescents and parents pre- and post-patch application in both conditions (M ± SE).

For RMSSD as a dependent variable, the 2 × 2 × 2 ANOVA showed a significant main effect of time (pre- to post-patch application, *F*(1,42) = 5.67, *p* = 0.022) with an increase in RMSSD, and a significant interaction of time × age group (*F*(1,42) = 14.05, *p* = 0.001) with an increase in both conditions in adolescents, but with nearly no change in parents (**Figure 1**). The main effect for condition and interactions of condition × age, condition × time, and condition × time × age were not significant (all *p* values > 0.05).

**Figure 2:**
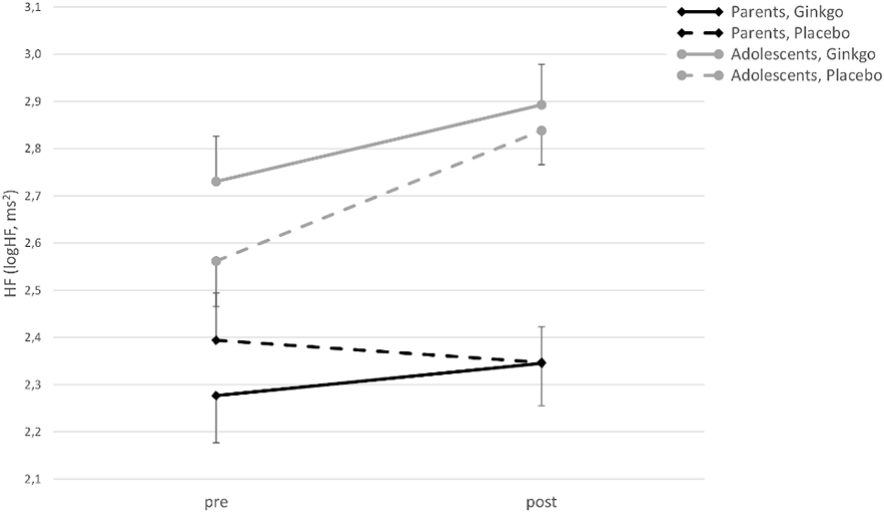
High-frequency power (HF) (logHF in ms2) in adolescents and parents pre- and post-patch application in both conditions (M ± SE).

For HF, there was a significant main effect of time (*F*(1,42) = 8.58, *p* = 0.005), an interaction of time × age group (*F*(1,42) = 7.04, *p* = 0.011), and an interaction effect of condition × time × age group (*F*(1,42) = 4.09, *p* = 0.049). **Figure 2** shows that HF increases from pre- to post-patch application in the Ginkgo condition in both adolescents and parents, but not in parents in the placebo condition. The main effect of the condition, and the interaction effects of condition × age group, and condition × time were not significant (all *p* values > 0.05).

**Figure 3:**
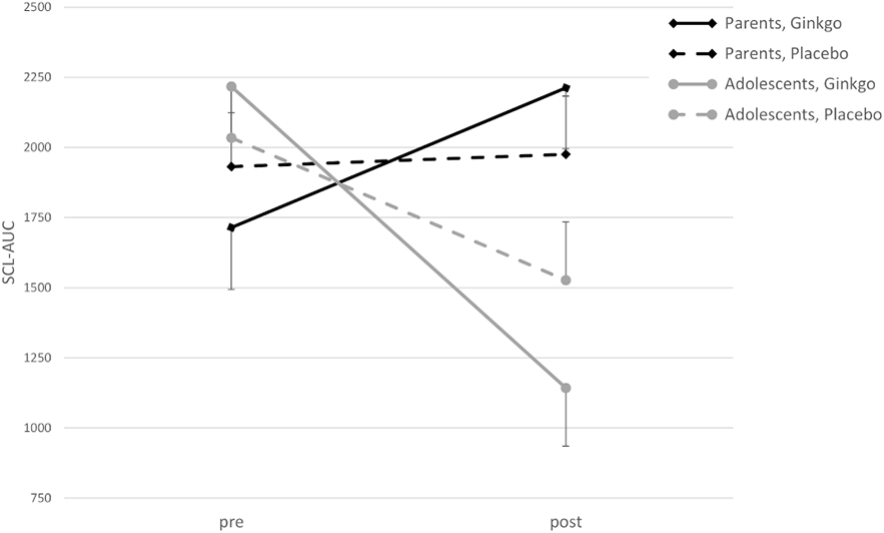
Skin conductance level–area under the curve (SCL-AUC) in adolescents and parents pre- and post-patch application in conditions (M ± SE).

For SCL-M there was a significant main effect of time (*F*(1,42) = 17.21, *p* < 0.001), and a significant time × age group interaction (*F*(1,42) = 4.65, *p* = 0.037). Furthermore, there were significant interaction effects of time × age group (*F*(1,42) = 8.61, *p* = 0.005) and condition × time × age group (*F*(1,42) = 4.44, *p* = 0.041) for SCL-AUC, with a decrease from pre- to post-patch application in both conditions in adolescents, but with nearly no change in the placebo and an increase in the Ginkgo condition in parents (**Figure 3**).

## Discussion

The aim of the present study was to experimentally induce placebo effects on cognitive performance and mood in healthy parent–child dyads. In a within-subjects design, placebo effects shall be induced through the application of a non-effective patch on the hips of participants accompanied either by the information that it is a Ginkgo patch which improves cognitive performance or by the information that the patch is a placebo only. In both conditions cognitive performance was measured by a PGNG test (34) and CVLT (33) while mood was assessed with POMS (13). Additionally, HRV and SCL were assessed as physiological stress markers.

Expectations about the effects of a Ginkgo patch on concentration, reaction times, memory, and mood ranged between neutral and high on a VAS from −50 to +50. They did not differ between adolescents and parents, and only correlated between adolescents and parents concerning reaction times. Additionally, parents’ expectations and adolescents’ placebo effects were associated with regard to reaction times in one of three levels, but this correlation did not withstand *p* value adjustment for multiple testing. It could be speculated whether adolescents’ expectations mediate the effect of parents’ expectations on adolescents’ placebo effects. Furthermore, there was only one significant correlation between expectations and placebo effects in parents which also did not withstand *p* value adjustment. Therefore, explicit expectations prior to the intervention did not affect the results.

Concerning the eight parameters of the PGNG, the only significant main effect of the within-subjects factor patch condition (information) could be found in the percentage of the correct target trials (PCTT) in level 3, paradoxically with a lower percentage in the Ginkgo condition compared to the placebo condition demonstrating a worse cognitive performance. The main effect of the between-subjects factor of age seems to be more constant, with significantly faster reaction times (RTT) in all three levels for adolescents. The significant interaction between the patch condition and the age group for PCIT in level 2 is noteworthy since there is a higher difference between means of PCIT in the Ginkgo than in the placebo condition in adolescents compared to adults. Moreover, this effect supports the hypothesis that adolescents have better cognitive inhibition performance with Ginkgo compared to the placebo condition, and therefore is the only placebo effect found in this study. Interpreting the data further, it seems likely that adolescents in general tend to react faster and more accurately, but their ability to inhibit reactions is inferior to that of adults. The age effect on reaction time is not surprising, as several studies report a decrease of reaction time with the process of ageing at least until young adulthood (40). Better inhibitory skills in adults in comparison to adolescents are a common finding which can be also interpreted in line with differences in functional-neural maturation (40, 41). Furthermore, reaction times, correct target, and inhibited trials might be interconnected to a certain degree. For example, subjects who take more time to respond to targets might respond more accurately to targets and vice versa. Our data, however, showed that there could be significant changes in one entity without significant changes in the other. Results of CVLT as a dependent variable showed no significant effects at all, neither for patch condition nor age.

Following the trend of the placebo effects on cognitive performance, no significant main effects of the factors “patch condition” or “age” could be observed for mood, as measured by the POMS pre–post-patch application differences in the ANOVAs. However, adolescents reported significantly better mood in response to the Ginkgo compared to the placebo patch. they also subjectively reported that the Ginkgo patch influenced their mood, at least according to the unadjusted p values. Furthermore, parents thought that the Ginkgo influenced their concentration when compared to the placebo patch.

The two examined parameters of HRV represent vagal influence on the heart function. Thus, a rise of both RMSSD and HF from baseline to post-patch application can be interpreted as a decrease of stress. For both parameters, there was a main effect of time but no significant main effect of the factor patch condition. Additionally, an interaction shows a stronger increase in adolescents for both. For HF, a three-way interaction could be found, indicating that adolescents show an increase in both conditions, whereas parents show an increase with the told Ginkgo condition, but a decrease in the told placebo condition. In contrast, SCL parameters indicate sympathetic activation and mirrored the effects on RMSSD and HF. Sympathetic activation decreased in adolescents in both conditions, with a stronger decrease in the Ginkgo condition, but increased in parents in the Ginkgo condition whereas there was no change in the placebo condition. Thus, adolescents responded in the hypothesized way and showed an increase in parasympathetic and a decrease in sympathetic activation in response to a putative active intervention.

Analyzed together, we could find a significant placebo effect in only 1 (PCIT level 2) out of 11 parameters for cognitive performance and in 1 (subjective mood) out of 6 parameters for mood and subjective assessment in adolescents. Additionally, there is one paradox effect for patch condition on PCTT level 3, which is hard to interpret. However, psychophysiological data show that, there is a significant reaction to the intervention itself, which is indicated by a rise of RMSSD and HF and a decrease in SCL particularly in adolescents who seem to be more sensitive to psychophysiological changes. The shown physiological reaction after the patch could be a base for placebo effects on cognitive performance and mood, which may not have shown up due to possible theoretical reasons as well as limitations of the study. These will be discussed in the following sections.

As mentioned in *Introduction*, the placebo effect in the context of analgesia is a well replicated phenomenon (1). Even in adolescents it was possible to experimentally induce placebo effects in the context of analgesia (25, 26). In the context of cognitive performance, the experimental induction of placebo effects may not be as easy to perform as analgesia or possibly just under special circumstances (25, 26). The lack of a placebo effect supports other findings that also could not induce placebo effects on cognition in a paradigm with methylphenidate which also used subjects, who have had no experience with this substance. However, a significant improvement of subjective mood and arousal through a placebo effect was reported (14). Other studies could not find placebo effects on cognition in coffee users which was induced by variety of information about decaffeinated coffee, although they found that the wrong information about real coffee worsens cognitive performance. Furthermore, there were no clear findings on mood improvement (15). Further studies did not find any placebo or nocebo effects on cognitive performance caused by altering the information when drinking real coffee (17). Together with the results from our study and those from the comparable exemplary studies reported, it can be argued that in order to approach the essence of a possible placebo effect on cognition and mood, some crucial aspects must be considered. First of all, substance users or non-users should be examined as this seems to have an effect. Substance users actually have an idea of how the substance’s effect should feel, whereas non-users do not have these experiences and have to link their expectations to theory, unbeknownst to the desired effect.

The well-replicated and easily inducible placebo effects in pain reduction might be due to a clear notion of what the desired effect should be—namely a pain reduction which has been experienced by every individual—often in the context of a painkiller. In line with this assumption, an experimental study showed a positive relationship between experienced pain relief during a preceding conditioning session and the later actual placebo effect in children, but not in adults (25). Adults seem to have a more robust history of pain reducing experiences than children. In contrast to analgesia—as a decrease of a specific symptom—the improvement of cognitive performance and mood could be a more unspecific and rare experience which is difficult to enumerate by healthy adults and adolescents. This could be a reason why placebo effects were harder to induce in these entities. The same argument concerning the amount and specificity of experiences apply, when thinking about the comparison of placebo effects in healthy subjects versus patients. The significance of several factors concerning the placebo effect in children and adolescents has previously been emphasized, such as the duration of disease, symptom severity and comorbidities (27). In adults, adolescents, and children suffering from diseases, it might be easier to induce placebo effects, because the expected effect is always towards a well-defined state of health or normality. In healthy people, however, the effect obviously must be some kind of “extra improvement.” Thus, it is easy to explain that concerning placebo effects on cognitive performance, large effects in clinical studies, for example in ADHD patients (21), can be found. The same has been shown for placebo effects on mood: There are many well replicated clinical findings about mood improving effects in treatment of depression (3, 10)—a pathological state of emotionality with a clear notion of a comparable healthy state. On the other hand, however, in experimental studies with healthy subjects as ours, and similarly to other studies, placebo effects on cognitive performance and mood cannot be induced or only under certain circumstances. Concerning placebo effects on cognitive performance, recent studies have focused on the role of expectancies about the effectiveness of the intervention (*post hoc* subjective outcome). In some cases, rather the expectancies affect objective cognitive performance than the sole information of receiving an intervention (42–44). High prior expectations can increase *post hoc* expectancies about the intervention, yet they do not necessarily affect objective cognitive outcomes (45).

### Limitations and Future Research

Some limitations of our study should be mentioned and discussed. First of all, it is not clear if the subjects really understood or internalized the effect of the different patches, despite forming mostly positive expectations of the Ginkgo effect. Although having been told about its positive effects it is possible that the effects have to be formulated in a more explicit and concrete way, e.g. improving reaction time, improving capability to memorize words, feeling happier, rather than talking about abstract entities as improving concentration, improving memory and mood. Maybe the subjects could not relate the tasks to the promised improvements. This assumption is supported by the lack of correlations between prior expectations and objective parameters and no differences between conditions in *post hoc* assessed subjective outcomes. Also, we did not explicitly ask subjects to rate their expectations about the effects of the placebo patch.

Furthermore, the usage of a transdermal patch for substance application is not common in our tested population. The time for an effect to take place was announced as 20–30 min in the experiment which might be not enough to mentally process the presence of the patch and consequently experience placebo effects. Although the subjects had positive expectancies about the substance itself, they might have been doubtful about an effect in such a short time period. To control for the effects of the application of a patch as an intervention, further studies should include a control group without any intervention or compare a patch application to other kinds of interventions such as pills or ointments. Additionally, due to our small sample size we did not explore the effects of different developmental phases or gender in children and adolescents or gender interactions with their accompanying parent. Finally, the PGNG and the CVLT might not be sensitive enough to detect differences between our conditions as they both might have been too easy which resulted in too little differences and a ceiling effect.

Due to the relative novelty of the paradigm, some aspects have to be optimized for future studies on placebo effects on cognitive performance and mood. These optimizations should include a correct and convincing induction of expectancies, an effective, salient application of the placebo substance, and an adequate allotted time period for the placebo effects to develop. The placebo could be more successful using common application forms, like pills, rather than transdermal patches. Moreover, from a theoretical point of view, a background of experience with the (placebo) substance or at least a concrete notion of how an effect should feel could be necessary for effective of placebo effects. Consequently, in experimental trials, a placebo sold as a familiar substance could be more effective, especially in subjects with a lot of experience with the substance in everyday life. Similarly, placebo effects on cognitive performance and mood might be easier to induce in subjects with such deficits because, in contrast to healthy subjects, an improvement towards a more concrete state is prospective. Thus, experimental trials on placebo effects on cognitive performance and mood in children or adolescents could also be conducted with subjects suffering from depression or attentional disorders. Aside from children, maybe elder people, who start to develop cognitive deficits in the form of mild cognitive impairment, could be a good target group in order to experimentally induce placebo effects on cognitive performance. Our limitations show that there are several other points that should be further investigated in future studies such as different developmental phases in cognitive development, gender differences, effects on varying aspects of cognitive performance, and a reasonable decision for the cognitive tests.

## Conclusion

In summary, we could not induce significant placebo effects on cognitive performance and mood in adolescents and their parents. This could particularly be due to some aspects of the study design such as the unusual form of application (transdermal patch) and substance used (Ginkgo) coupled with the fact that it could not work in health subjects without cognitive impairment or mood disturbances. However, we could show that adolescents are more sensitive to psychophysiological reactions to interventions—if they work or not—than adults, and this could be part of the underlying mechanism of placebo effects.

## Author’s Note

This study was part of DW’s dissertation (Watolla D. Placebo effects on cognitive performance and mood in children and parents—an experimental approach [unpublished dissertation]. [Tübingen, Germany]: Eberhard Karls University; 2017. 75 p.).

## Data Availability Statement

The raw data supporting the conclusions of this article will be made available by the authors, without undue reservation, to any qualified researcher.

## Ethics Statement

The studies involving human participants were reviewed and approved by the Ethical Review Board of the University of Tübingen. Written informed consent to participate in this study was provided by the participants and the participants’ legal guardian/next of kin.

## Author Contributions

PE, KW, and DW contributed conception and design of the study. DW performed the study. DW and KW organized the database, performed the statistical analysis, and wrote the first draft of the manuscript. JS-K and HS contributed design features concerning the inclusion of adolescents. NM and SG contributed design features and analyses of psychophysiological data. NM, SG, and MG wrote sections of the manuscript. All authors contributed to manuscript revision, read, and approved the submitted version.

## Funding

This work was supported by grants of the Fortüne-Program of the University of Tübingen (fortüne 2179-0-0 and 2266-0-0) for KW. We acknowledge support by Deutsche Forschungsgemeinschaft and Open Access Publishing Fund of the University of Tübingen. JS-K was supported by a grant from the Faculty of Medicine, Tübingen (TÜFF no. 2399‐0‐0).

## Conflict of Interest

The authors declare that the research was conducted in the absence of any commercial or financial relationships that could be construed as a potential conflict of interest.

The handling editor is currently co-organizing a Research Topic with one of the authors PE and KW, and confirms the absence of any other collaboration.
